# Thigmotaxis Mediates Trail Odour Disruption

**DOI:** 10.1038/s41598-017-01958-z

**Published:** 2017-05-10

**Authors:** Lloyd D. Stringer, Joshua E. Corn, Hyun Sik Roh, Alfredo Jiménez-Pérez, Lee-Anne M. Manning, Aimee R. Harper, David M. Suckling

**Affiliations:** 1 The New Zealand Institute for Plant & Food Research Limited, PB 4704, Christchurch, 8140 New Zealand; 2 Division of Applied Life Science (BK21+ program), Gyeongsang National University, 501 Jinju-daero, Jinju, Gyeongnam, 660-701 Republic of Korea; 3 Laboratorio de Ecología Química, Centro de Desarrollo de Productos Bióticos, Instituto Politécnico Nacional, Calle Ceprobi No. 8, San Isidro, Yautepec, 62731 Morelos Mexico; 4 Better Border Biosecurity, Auckland, New Zealand; 5 School of Biological Sciences, University of Auckland, Tamaki Campus, Building 733, Auckland, New Zealand

## Abstract

Disruption of foraging using oversupply of ant trail pheromones is a novel pest management application under investigation. It presents an opportunity to investigate the interaction of sensory modalities by removal of one of the modes. Superficially similar to sex pheromone-based mating disruption in moths, ant trail pheromone disruption lacks an equivalent mechanistic understanding of how the ants respond to an oversupply of their trail pheromone. Since significant compromise of one sensory modality essential for trail following (chemotaxis) has been demonstrated, we hypothesised that other sensory modalities such as thigmotaxis could act to reduce the impact on olfactory disruption of foraging behaviour. To test this, we provided a physical stimulus of thread to aid trailing by Argentine ants otherwise under disruptive pheromone concentrations. Trail following success was higher using a physical cue. While trail integrity reduced under continuous over-supply of trail pheromone delivered directly on the thread, provision of a physical cue in the form of thread slightly improved trail following and mediated trail disruption from high concentrations upwind. Our results indicate that ants are able to use physical structures to reduce but not eliminate the effects of trail pheromone disruption.

## Introduction

The Argentine ant, *Linepithema humile* (Mayr) is widely distributed across the globe^1^, with populations in Europe^2^, California^3^, Australia^4^, Japan^5^ and New Zealand^6, 7^. Argentine ants are an invasive species^8, 9^ posing an agricultural risk through farming of destructive sap sucking Hemiptera^10^, as well as competing with other invertebrate species such as pollinators^11^ and seed dispersers^12^ for food. Their extensive distribution has been attributed to their association with humans^13^, and their success in their new habitats has been related to a superior colony size leading to numerical dominance over resident ant populations^14–17^.

Argentine ants are reliant on chemical cues for orientation and food retrieval^18, 19^. Their heavy reliance on trail pheromones as a predominant mode of orienting and dominating a landscape^20^ may also be their Achilles heel^21^. The use of the trail pheromone (*Z*)-9-hexadecenal for Argentine ant management has recently been examined as an environmentally benign control method for this species^22–27^. Pheromone-based pest management offers the advantages of low-hazard and high target specificity, avoiding many unwanted off-target effects^28^. The use of trail pheromone disruption for the Argentine ant as a new control method is based on the successful moth mating disruption paradigm already in use^29, 30^, and by coincidence of chemistry also involves a straight chain lepidopteran sex pheromone that is available commercially.

The mechanism, concentrations, and factors affecting trail following in Argentine ants were examined by van Vorhis Key *et al*.^31^ and van Vorhis Key and Baker^32–34^, based on the earlier work of Cavill *et al*.^35^ who made extracts from Argentine ant ventral glands. They quantified the (*Z*)-9-hexadecenal content, acquiring approximately 300 ng of (*Z*)-9-hexadecenal from 370 individually dissected ventral glands. Van Vorhis key and Baker^33^ compared their synthetic (*Z*)-9-hexadecenal activity and persistence to that of a total gaster extract and reported a 200-fold negative difference in activity of synthetic pheromone compared to that of the gaster extract. They also reported a marked difference in longevity, with gaster extract trails eliciting trail following behaviour significantly longer than synthetic (*Z*)-9-hexadecenal trails. This work corroborated Cavill *et al*.^35^ who reported forager ants preferring gaster extract to synthetic (*Z*)-9-hexadecenal in multi-choice trail-following trials. Further research has highlighted that additional compounds may be used for trail following in *L. humile*, yet a greater concentration of those compounds is required to induce trail following behaviour than when (*Z*)-9-hexadecenal is used alone^36^.

In outdoor populations, we have observed that Argentine ants often trail along edges or the boundary of two objects (thigmotaxis), suggesting that tactile cues may be employed during trail following. This behaviour has also been demonstrated in laboratory populations of *Lasius niger* whereby ants predominantly chose to follow a bridge with a wall over a bridge without a wall when offered the choice in the absence of any chemical cues^36^. We hypothesised that if tactile cues were present, then trail pheromone disruption using (*Z*)-9-hexadecenal would be more difficult to achieve, requiring a higher concentration of pheromone for complete disruption or that in the presence of this cue, complete disruption may not be achievable. We expected a beneficial interaction from the presence compared with absence of a physical cue against a background of trail odour. We set out to test how trail pheromone concentration released from a physical substrate affected trailing ability under natural conditions (thigmotaxis enhances trailing). We also tested the situation where disruption of trailing might be achieved less effectively in the presence of physical cues (we theorised that thigmotaxis enhances trailing, reducing disruption). We followed the work by Cavill *et al*.^35^ and Van Vorhis Key and Baker^33^ and used the methods of Suckling *et al*.^23^ to digitise tracks in order to compare the trail following behaviour to gaster extracts and synthetic (*Z*)-9-hexadecenal, and trail following ability under pheromone disruption with a countervailing physical cue. We used cotton thread to absorb and release the trail pheromone and enable the two sensory modalities (olfaction and thigmotaxis) to be neatly examined independently and together. We applied the trail integrity statistic (r^2^), derived from linear regression from digitised trails.

## Results

### Experiment 1

#### Trail following with gaster extract and synthetic (*Z*)-9-hexadecenal

From the GC-MS analysis of gaster extracts we determined that there was 3.7 ± 0.65 ng (mean ± SEM) of (*Z*)-9-hexadecenal per worker gaster (range 5.4–2.3 ng/gaster), above the previously reported 0.81 ng/gaster^35^. The chromatoprobe processing of whole ants yielded significant variation in the amount of (*Z*)-9-hexadecenal detected for each ant. (*Z*)-9-hexadecenal levels were below the detectable limit of the GC-MS for two of the ants, median amount 4.7 ng, mean 8.4 ng, maximum 21.8 ng.

**Figure 1:**
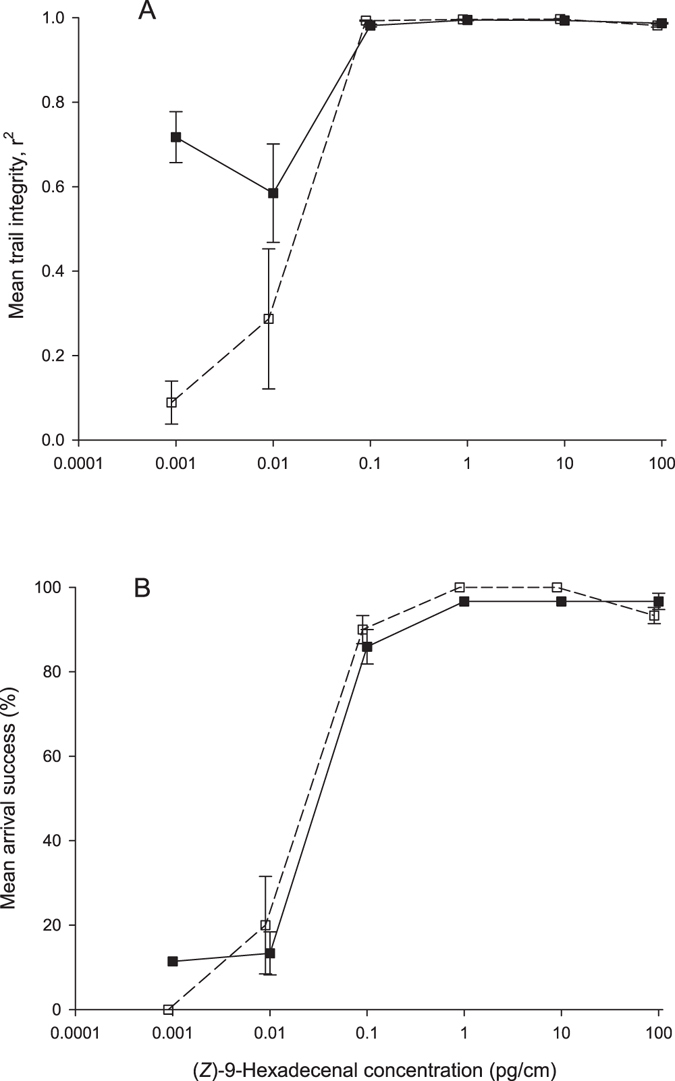
Trail following measured by trail integrity statistic (**A**) and percentage arrival success to food (**B**) of the Argentine ant *Linepithema humile* to dilutions of total gaster extract (□) or dilutions of synthetic (*Z*)-9-hexadecenal (■). Error bars show one standard error.

Trailing of ants to gaster extract dilutions occurred across four orders of magnitude between 0.1 and 100 pg/cm, with recorded trail integrity r^2^ values over 0.97 (Fig. 1A). Percentage arrival success at the sugar solution was similarly above 90% across those concentrations (Fig. 1B). Low r^2^ and arrival success were observed at 0.009 pg/cm (r^2^ = 0.29 and 20% arrival success). No trail following behaviour observed for the gaster extract at 0.001 pg/cm (Fig. 1A).

**Figure 2:**
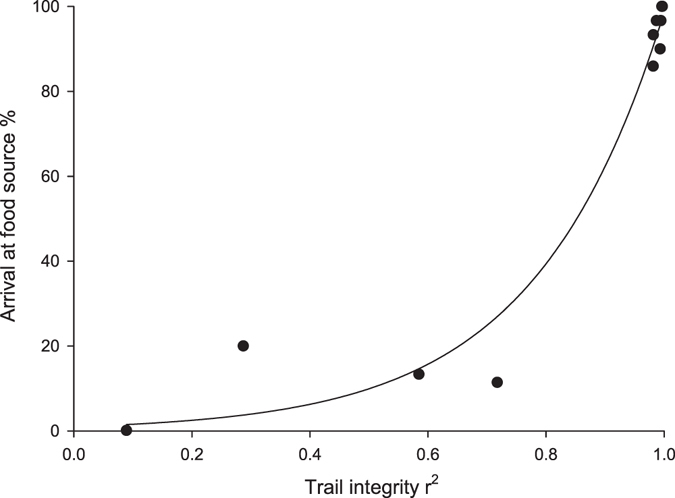
Correlation between Argentine ant *Linepithema humile* trail integrity and foraging success with an exponential function from pooled gaster extract and synthetic trail results, Y = e^4.5913x^, r^2^ = 0.9684.

We found that synthetic (*Z*)-9-hexadecenal elicited trail following at similar concentrations to gaster extract with trail following occurring between 0.01 and 100 pg/cm. The two lowest concentrations achieved arrival success below 20% (Fig. 1B), and arrival success was comparable to the two lowest gaster extract concentrations. The correlation between trail integrity and recruitment success followed an exponential function Y = e^4.5913x^ (r^2^ = 0.9684) (Fig. 2). Using that function, arrival success is expected to drop to 50%, once the r^2^ value declines to 0.85.

### Experiment 2

#### Disrupting thigmotaxis with odour

**Figure 3:**
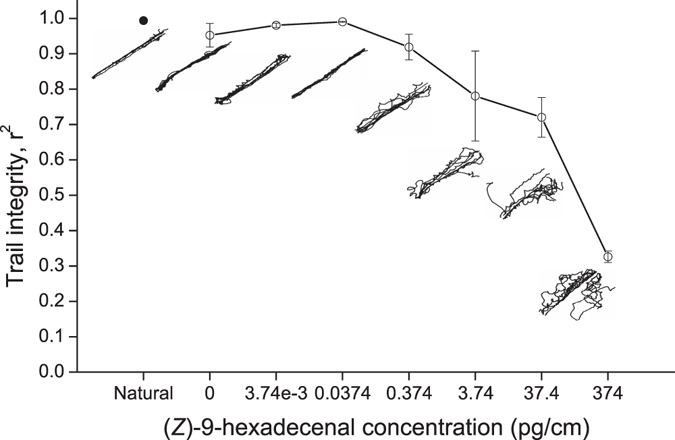
Trail integrity (r^2^) for the Argentine ant *Linepithema humile* when trailing on a 20 cm length of thread either containing naturally laid trail pheromone (natural control [●]), untreated or dipped in increasing trail pheromone (*Z*)-9-hexadecenal concentrations (○). Typical tracks are displayed for one replicate from each treatment. Error bars show one standard error.

Trail integrity r^2^ of 0.95 was achieved in the absence of synthetic (*Z*)-9-hexadecenal when only ethanol had been applied to a piece of thread and evaporated (Fig. 3). The scatter in trail integrity, r^2^, in the control-thread treatments was greater than for individuals following naturally-deposited trail pheromones on thread (r^2^ = 0.99). However, a decrease in trail integrity was observed when synthetic trail pheromone concentrations on the thread were greater than 0.374 pg/cm, with a low value (r^2^ = 0.33) in the 374 pg/cm treatment (Fig. 3). These large scale effects were evident in the digitised trails of individual ants (Fig. 3).

### Experiment 3

#### Effect of thigmotaxis on trail disruption

**Figure 4:**
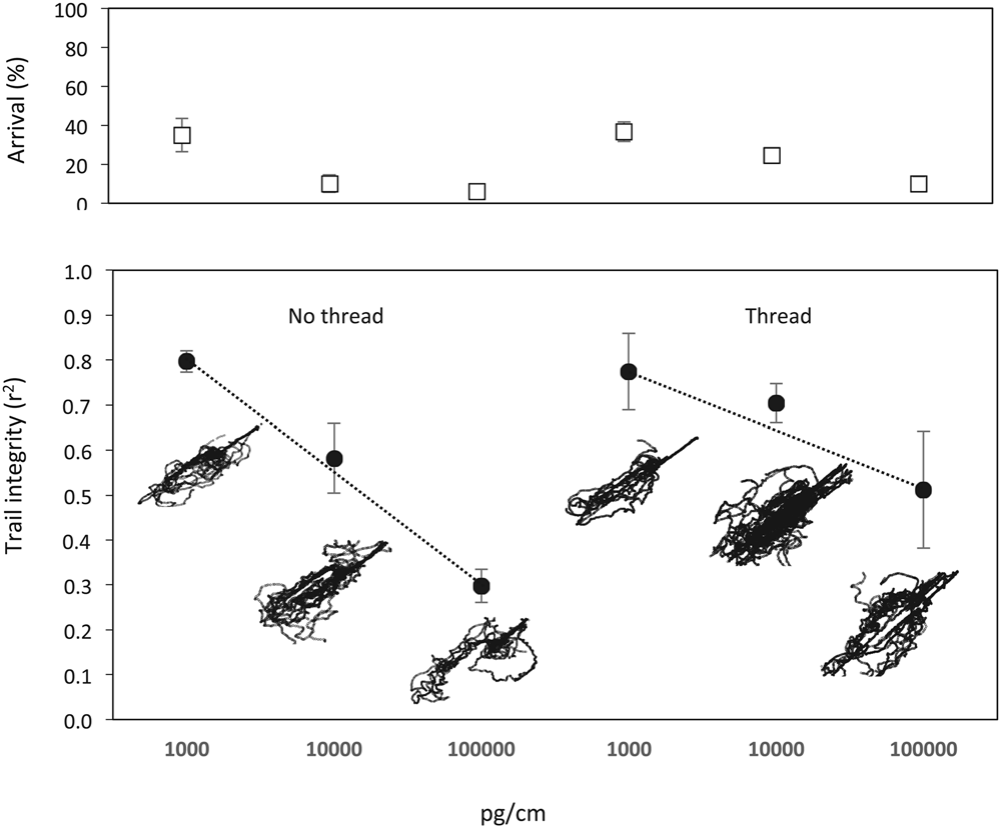
Trail following behaviour of *Linepithema humile* on a 1 pg/cm trail with and without thread present, located 1 cm downwind from three high rate trails of (*Z*)-9-hexadecenal on glass, no thread data re-analysed from^41^. Typical tracks are displayed for one replicate from each treatment. Error bars show one standard error.

Excess pheromone was deposited with a capillary on glass and a piece of thread was added as a physical cue over the top of the downwind 1 pg/cm trail. For the main effects, the increase in pheromone concentration reduced trail following integrity (F_(2,18)_ = 15.93; P < 0.001), and the presence of thread somewhat reduced the pheromone disruption effect (F_(2,18)_ = 3.34; P = 0.084). The slope of the thread versus no thread regression models of the r^2^ values for each replicate indicated that loss of trail integrity under pheromone disruption in the presence of thread was occurring, but almost half that of trails under pheromone disruption without a physical aid present (Fig. 4). However, this difference in trail integrity for the interaction term for pheromone concentration and the presence of thread was not significant (F_(2,18)_ = 1.24; P = 0.313). Arrival success rate was not different between trials with and without thread (Z = 0.04, P = 0.967) for the full model. While trail integrity was higher in the presence of the physical aid, the physical cue did not override the trail disruption from excess chemical pheromone. The highest trail integrity values with or without thread were ~80% integrity at 1000 pg/cm, but this dropped to ~30% integrity without thread, compared with ~50% with thread present.

## Discussion

When no synthetic pheromone was present on thread, the ants followed the thread closely (r^2^ = 0.95), suggesting the use of tactile cues could influence the initial spatial deposition of trail pheromone when ants are first laying trails. When pheromone-dipped thread of different concentrations was offered to the ants to test trail following behaviour in the presence of synthetic trail pheromone, it was observed that trail integrity was high (r^2^ > 0.90) over three orders of magnitude of trail strength. Despite the presence of thread for thigmotaxis, odour still appeared to be the ant’s primary sensory mode as there was a reduction in trail integrity at the top odour concentrations. At a high loading of pheromone on the thread (>3.74 pg/cm), trail integrity (r^2^ = 0.78) was already at a threshold at which ants would have a reduced ability to successfully recruit to a food source (Fig. 2). The (*Z*)-9-hexadecenal concentration range that we found to elicit trail following is in concordance with that of other known ant trail pheromone systems^37^. Ants trailed along gaster extract dilutions across four orders of magnitude between 0.09 and 90 pg/cm, or 0.00075 to 0.75 gaster equivalents. This is similar to the results of Van Vorhis Key *et al*.^31^ who reported optimal trailing at 0.1 and 1.0 gaster equivalents and some minor trail following response down to 10^−4^ gaster equivalents. In contrast to previous findings, we found that synthetic (*Z*)-9-hexadecenal elicited trail following at similar concentrations to gaster extracts, unlike the other studies that required 100-fold higher concentration of synthetic (*Z*)-9-hexadecenal (98% purity) to elicit similar trail following to a gaster extract^33, 35^. This could be related to differences in methodology and purity of synthetic (*Z*)-9-hexadecenal. The ability of ants to follow (*Z*)-9-hexadecenal at 0.001 pg/cm was surprising and was better than trail following to the gaster extract at 0.0009 pg/cm. However, trail integrity was still poor and only 11% of ants arrived to the food source at the end to the trail. Van Vorhis Key and Baker^32^ suggested that additional components are likely to play a role in the Argentine ant trail pheromone system and the ability of the Argentine ant to follow trails consisting of alternative compounds that have been detected from their natural trails, (dolichodial and iridomyrmecin), support this. Curiously, the ants appear to be less sensitive to these compounds^38^, which are not commercially available, and did not improve trail following and arrival success rates.

Despite a high upwind pheromone loading and low trail integrity when thread was placed on top of trail pheromone deposited with a glass capillary, ants were observed responding to the trail. This was often at a distance of 1–2 cm with repeated bouncing off the thread observed like a scalloping line, suggesting a tactile behavioural response emerging when trail following ability has been impeded by an oversupply of (*Z*)-9-hexadecenal. This unusual behaviour of ants following a trail 1 cm downwind from a strong trail, may be due to the ant sensing the maximum natural pheromone threshold beyond which concentration gradient of the odour plume perpendicular to the thread fades out^39^. Ants use this gradient to orient left and right along a trail. Thus, when too close to the odour source, the ants are disorientated. The tactile cues from the thread may have allowed them to re-orientate. However, at excess (*Z*)-9-hexadecenal concentrations seen as area-wide trail disruption, the odour may diffuse to such an extent that trailing ability is disrupted on both the outward and return journey. This was demonstrated in red imported fire ants, *Solenopsis invicta*, when outward and return journeys were analysed separately in the presence of excess trail pheromone^40^.

The presence of thread appeared to only slightly improve orientation ability under excess pheromone but not mitigate the effects from an oversupply of pheromone, supporting Aron *et al*.^19^ who determined that Argentine ants predominantly use olfactory cues for orientation. It is not known whether Argentine ants can overcome trail pheromone disruption by using other cues, but now this can be explored because the primary channel can be non-destructively removed by merely adding an excess of the synthetic trail pheromone. The ants do not appear to switch from olfactory to solely thigmotatic mode of foraging in the presence of an over-supply of (*Z*)-9-hexadecenal. However, their ability to recover quickly from an over-supply of the pheromone and follow a pheromone trail when they are in clean air^41^ suggests that disrupting them in windy situations would be harder than in areas with low wind velocities. It is possible to see some straight line behaviours in the generally disrupted behaviours, although the r^2^ statistic appears to follow the dose response better than the overlaid trail images might suggest. A reduction in their ability to effectively communicate in areas under long-term control using trail pheromone disruption would probably allow other ant species to secure more resources^21^. This reduction in food availability would have a negative impact on colony size, further reducing their competitive ability and nest stability over time, but requires treatment over a broad scale. Our results indicate that further issues could arise for the approach of trail disruption when ants are able to use physical structures to overcome sensory overload and partially overcome disruption. This could be more likely in the built environment, where these ants are known to do well^6^.

## Methods

### Insects

Five queen-right Argentine ant colonies of similar size based on worker ant activity and with all brood life stages present were used. Colonies were field collected from Christchurch, New Zealand 43°34 S 172°35E in December 2009, April and November 2010. Colonies had access to water but were starved of protein and carbohydrates 2–3 days prior to experiments to encourage foraging during assays.

### Chemicals

The (*Z*)-9-hexadecenal was purchased from Bedoukian Research (Danbury, CT, USA) (90% chemical purity).

### Experiment 1

#### Trail following with a gaster extract and synthetic (*Z*)-9-hexadecenal

Argentine ant gaster extracts were made by excising whole gasters from frozen worker ants. Four replicates of 50 gasters each were submerged in 200 µl of n-hexane containing 50 ng of dodecyl acetate as an internal standard for 30 minutes, after which the solvent was drawn off. The (*Z*)-9-hexadecenal content was quantified by Gas Chromatography coupled Mass Spectrometry (GC-MS) analysis. In addition, the amount of (*Z*)-9-hexadecanal per worker was assessed by placing single ants immobilised by freezing for ca. 10 minutes into a ChromatoProbe quartz microvial 15 mm long, 2 mm i.d. Microvials were placed into the injector which had been fitted with a ChromatoProbe kit for a Varian 3800 GC coupled to a Saturn 2200 MS system (Varian Associates, Inc., Walnut Creek, CA, USA). This kit allows the thermal desorption of small amounts of solids or liquids contained in quartz microvials^42^. A chromatoprobe has the advantage that the whole ant is sampled rather than an extract of a sample that is likely to still have some of the compound remain behind. Quantification was by external standard method whereby, 1 µl of a 10 ng/µl solution of (*Z*)-9-hexadecenal was added to a separate microvial and placed into the chromatoprobe. The injector was programmed at 40 °C for 1 minute then heated to 250 °C at a rate of 200 °C/min and held there for 2 minutes to allow desorption of the compounds from the ants. The split ratio of the injector was initially set at 50 then at 0.5 minutes changed to splitless, then at 1 minute the split ratio was set at 10. The GC oven was programmed at 40 °C for 1 minute then heated to 250 °C at 6 °C/min, then further heated to 260 °C at 10 °C/min and held there for 10 minutes. The retention time, and peak area for the (*Z*)-9-hexadecenal external standard solution was used to determine the amount of (*Z*)-9-hexadecenal per worker. All MS analyses were done in electron impact mode using an ionisation voltage of 70 eV, and a mass range of 29 m/z to 399 m/z. The limit of detection of the instrument for (*Z*)-9-hexadecenal was estimated to be >0.1 ng.

The experimental setup was similar to that illustrated earlier in supplementary material^41^ whereby a nest was connected to a glass plate (400 × 200 × 5 mm) via a wire bridge, and ants were allowed to discover and forage at a food naturally for 1 hr prior to each trial. At the beginning of the trials, the glass plate was replaced with a fresh clean glass plate, and any ants on the used glass plate brushed back into the source colony. A piece of paper with a faint pencil-ruled line was placed under the glass plate to support the application of a straight line of treatment pheromone. A 5 μl glass capillary was used to lay a 30 cm long trail of the gaster extract solution on the glass from the wire bridge to a 30% sugar solution-soaked cotton dental roll, immediately before the wire bridge was connected to the colony. The dental roll was used to prevent ants returning to the nest and sharing information or depositing addition pheromones on the way back to the colony during the trials. Six gaster extract dilution treatments in n-hexane were trialled (0.0009, 0.009, 0.09, 0.9, 9 and 90 pg/cm). This was replicated three times with 10 ants recorded per treatment. For each replicate and treatment a new nest and clean glass was used and the wire bridge was cleaned with an n-hexane wipe. Trials were conducted between 9 am and 3 pm (2–3 trials per day) and a nest was only used once per day and only used twice prior to being fed to preserve nest condition and reduce nest-escaping behaviour. After feeding, the colony was starved again prior to experiments. All trials were conducted at 21° ± 1 °C. As a fume hood was in operation to extract excess pheromone from the room, the trails were oriented in the same direction of airflow, with the nest positioned upwind and the end of the trail where the sugar water-cotton was positioned downwind. The airflow at the middle of the trail was below the detectable limit of the hotwire anemometer (YK-2005AH Lutron Electronic Enterprise Co. Ltd., Taiwan), however, 35 cm from the sugar water-cotton end of the trail towards the fume hood the airspeed was 0.1 m/s. Ant movements (n = 10 per treatment) were recorded overhead with a Logitech^®^ webcam and the subsequent video recordings analysed in MaxTRAQ V1.92 (Innovision Systems Inc.) to give individual digitised trails^23^. The trails were oriented at a 45° angle to ensure maximum length for measurement of trail integrity (r^2^). An r^2^ value near 1.00 indicates perfect trail integrity or linearity; good trail integrity forms a comparatively straight line. During poor trail following, ants can be spread out across the frame resulting in low r^2^ values^41^. The trail integrity statistic and percentage arrival success were used to characterise trailing activity. Gaster extract activity was compared to synthetic (*Z*)-9-hexadecenal activity (0.001, 0.01, 0.1, 1, 10 and 100 pg/cm).

### Experiment 2

#### Thigmotactic and trail pheromone cues combined

Lengths of cotton thread (20 cm × 0.33 mm) were submerged in different concentrations of (*Z*)-9-hexadecenal diluted in 70% ethanol (0, 0.01, 0.1, 1, 10, 100, 1000 µg/ml), and the solvent allowed to evaporate for one minute before trials. The thread was placed on glass as above and trials were conducted at 21° ± 1 °C.

To quantify the amount of pheromone absorbed onto the thread, 0031 metre of thread was dipped into a solution of (*Z*)-9-hexadecenal 70% ethanol at 100, 10 and 1 µg/mL for 10 seconds, with five replicates at each concentration. This was laid out to dry for one minute, and then soaked in 1 mL absolute ethanol containing 5, 0.5 or 0.05 µg of (*Z*)-9-tetradecenal respectively as an internal standard. After extracting the thread for 24 hours, the thread was removed and the extract analysed by GC-MS as above. Injections were splitless for 0.6 min and a constant flow of 1 ml per min of helium was used as the carrier gas. The oven was equipped with a DB-5 column with dimensions of 30 m × 0.25 mm i.d. × 0.25 µm film thickness, and programmed to hold at 40 °C for 2 min, then increase at 10 °C/min to 280 °C, where it was held for 15 min.

A solution of 70% ethanol was used as a solvent control. A natural trail control was tested by allowing ants to deposit trail pheromone on thread suspended tightly between the colony and a sugar water feeder for one hour. Ant-treated thread lengths (20 cm) were then placed on a horizontal glass plate (400 × 200 × 5 mm) with a white background for contrast. A sugar water wick placed at one end of the thread was used to assess success rates of ants following the pheromone to the food source, and to entice ants to stay rather than walking back down the thread. The other end of the thread was placed at the base of a wire bridge that connected the ant colony to the glass plate. Ants were digitally recorded and r^2^ determined as above. Ten ants were recorded for each of the five replicates for each treatment during their journey across the 20 cm distance between colony and sugar water (n = 30–50 ants trailing), but there was poor trailing in the 1000 µg/ml treatment, so only three replicates could be completed. The high concentration of pheromone of the thread led to apparent avoidance behaviour at the end of the wire, whereby ants were observed walking back down the wire. One of the ethanol control replicates was discarded as one of the r^2^ values (0.08) was an extreme outlier. It is not known why this occurred.

### Experiment 3

#### Effect of thigmotaxis on trail disruption

To determine whether ants could overcome trail disruption with the aid of a tactile cue, we modified the methods reported earlier^41^. Two parallel pheromone trails 1 cm apart were presented to the ants. One trail, the main trail, was connected to the wire bridge at one end and food at the other end as above, and a second trail, a shorter disruptive trail was deposited 1 cm upwind of the main trial. Both trails were drawn directly on the glass with a 5 μl glass pipette and the trail was oriented so that the wind flow was from the disruption trail to main trail and then evacuated by the fume hood. In this trial, the main trail (1 pg/cm (*Z*)-9-hexadecenal) was overlaid with a length of clean length of thread. The disruptive second trail, 1 cm upwind (wind speed was 0.1 m/s), had a concentration of either 1000, 10,000 or 100,000 pg/cm. Ten ants were recorded per treatment and each treatment was replicated three times. Trail integrity (r^2^) and arrival success (the percentage of ants that arrived versus ants that started on the thread, but wandered off and didn’t arrive at the sugar bait) was determined and results were compared to earlier reported experiments with this method without the thread.

### Statistical Analysis

The effect of thigmotaxis was tested by an ANOVA on the angular-transformed r^2^ values, and a binary logistic regression on arrival success. Slopes of trail integrity-dose responses with and without thread (providing a cue for thigmotaxis) were compared by ANOVA. All statistics were performed using Minitab 16.
